# Reward vs. Retaliation—the Role of the Mesocorticolimbic Salience Network in Human Reactive Aggression

**DOI:** 10.3389/fnbeh.2016.00179

**Published:** 2016-09-27

**Authors:** Gabriela Gan, Rebecca N. Preston-Campbell, Scott J. Moeller, Joel L. Steinberg, Scott D. Lane, Thomas Maloney, Muhammad A. Parvaz, Rita Z. Goldstein, Nelly Alia-Klein

**Affiliations:** ^1^Psychiatry and Neuroscience, Icahn School of Medicine at Mount SinaiNew York, NY, USA; ^2^Division of Social and Behavioral Sciences, Lindenwood University-BellevilleBelleville, IL, USA; ^3^Psychiatry, Virginia Commonwealth UniversityRichmond, VA, USA; ^4^Psychiatry and Behavioral Sciences, University of Texas Health Science Center at HoustonHouston, TX, USA

**Keywords:** reactive aggression, intermittent explosive disorder, point-subtraction aggression paradigm, reward, salience, mesocorticolimbic network

## Abstract

The propensity for reactive aggression (RA) which occurs in response to provocation has been linked to hyperresponsivity of the mesocorticolimbic reward network in healthy adults. Here, we aim to elucidate the role of the mesocorticolimbic network in clinically significant RA for two competing motivated behaviors, reward-seeking vs. retaliation. 18 male participants performed a variant of the Point-Subtraction Aggression Paradigm (PSAP) during functional magnetic resonance imaging (fMRI). We examined whether RA participants compared with non-aggressive controls would choose to obtain a monetary reward over the opportunity to retaliate against a fictitious opponent, who provoked the participant by randomly stealing money from his earnings. Across all fMRI-PSAP runs, RA individuals vs. controls chose to work harder to earn money but not to retaliate. When engaging in such reward-seeking behavior vs. retaliation in a single fMRI-PSAP run, RA individuals exhibited increased activation in the insular-striatal part of the mesocorticolimbic salience network, and decreased precuneus and ventromedial prefrontal cortex activation compared to controls. Enhanced overall reward-seeking behavior along with an up-regulation of the mesocorticolimbic salience network and a down-regulation of the default-mode network in RA individuals indicate that RA individuals are willing to work more for monetary reward than for retaliation when presented with a choice. Our findings may suggest that the use of positive reinforcement might represent an efficacious intervention approach for the potential reduction of retaliatory behavior in clinically significant RA.

## Introduction

Reactive aggression (RA) is an approach-related impulsive response committed in anger-provoking or threatening social situations (Carver and Harmon-Jones, 2009; Blair, 2012). The psychiatric disorder, intermittent explosive disorder (IED), is marked by recurrent verbal or physical outbursts of RA that are grossly out of proportion to the experienced provocation, affecting 7% of the US population (lifetime prevalence; Kessler et al., 2006; Coccaro et al., 2014). Although, repeated outbursts of RA in clinical or sub-clinical IED cause marked personal distress due to interpersonal, legal or financial problems, and are linked to vast societal costs arising from violent behavior (Kessler et al., 2006), the neurobiological underpinnings of RA are not well understood.

Neurobiological models suggest that RA in IED is mediated by a functional imbalance between prefrontal cortical “control” areas and mesolimbic “emotion” areas (Coccaro et al., 2007; Siever, 2008). In this model, there is increased reactivity of the bottom-up “drives” in mesolimbic regions including the amygdala and the insula to arousing emotional events, and impaired functioning of top-down “brakes” in regions including the anterior cingulate cortex (ACC), and the lateral and medial sections of the prefrontal cortex (PFC), areas implicated in anger regulation and impulse control (Davidson et al., 2000; Siever, 2008). This imbalance in the functioning of mesolimbic-prefrontal circuits can manifest as a failure of integration of social cues and self-regulation (Heatherton and Wagner, 2011), precipitating disproportionate RA (Buckholtz and Meyer-Lindenberg, 2008; Siever, 2008; Coccaro, 2012).

Neuroimaging studies in IED, borderline personality disorder, and alcohol use disorder, other clinical populations with high levels of RA, provide supporting evidence for the “bottom-up drives/top-down brakes” theory of human RA. For example, as compared to healthy controls, IED patients demonstrated increased amygdala reactivity and diminished amygdala-orbitofrontal cortex coupling during processing of social threat signals (i.e., angry faces; Coccaro et al., 2007). Moreover, individuals with borderline personality disorder and comorbid IED showed increased glucose metabolism in the amygdala and orbitofrontal cortex (measured with ^18^fluoro-deoxyglucose positron emission tomography [PET]) during provocation in the Point-Subtraction Aggression Paradigm (PSAP) (New et al., 2009), a well-validated paradigm to elicit RA by provoking participants with money subtractions (e.g., Cherek, 1981; Cherek et al., 1997, 2000). In an fMRI-adapted PSAP version, individuals with past alcohol use disorder showed increased aggressive and monetary reward-seeking behavior and exhibited decreased provocation-related activation in “top-down” PFC areas compared to controls (Kose et al., 2015).

Moreover, evidence from animal and human studies suggests an involvement of the striatum in the initiation, execution, and termination of aggressive behavior across species (Soderstrom et al., 2001; Ferrari et al., 2003; de Almeida et al., 2005; Nelson and Trainor, 2007; Seo et al., 2008; Yu et al., 2014). However, its exact role for aggression is not clear yet. For example, fMRI studies using the Taylor-Aggression Paradigm, a competitive reaction-time task performed against two fictitious opponents, have demonstrated that high levels of RA in healthy participants were linked to increased striatal reactivity (Krämer et al., 2011; Beyer et al., 2014; Gan et al., 2015), possibly indicating arousing or even rewarding properties of aggressive behavior. In contrast, high levels of RA in healthy men measured with the PSAP were linked to low resting dopamine synthesis (quantified with 6-[^18^F]-fluoro-_L_-DOPA-PET) in the striatum and the midbrain (Schluter et al., 2013). Another line of research targeting altered reward sensitivity as a phenotype for aggressive behavior shows that healthy adults with pronounced impulsive-antisocial psychopathic traits, which are associated with RA, exhibited increased ventral striatum reactivity to monetary rewards and amphetamine (Buckholtz et al., 2010), and that incarcerated criminals with pronounced impulsive/antisocial traits showed altered neural connectivity between the brain's reward system and the PFC (Geurts et al., 2016). These findings add to a recent proposition that individuals prone to impulsive-RA might share the neural circuitry such as impaired PFC control and enhanced reward sensitivity with other “compulsive” problem behaviors such as drug and behavioral addictions (Stahl, 2015). According to this proposition, impulsive-RA might be a deeply ingrained habit-like behavior that might be the consequence of stimulus-response learning between provoking situations and subsequent aggressive behavior, a process presumably associated with dysfunction of the dopaminergic mesocorticolimbic reward system. Thus, the role of the striatum in mediating the rewarding or arousing properties of RA (Couppis and Kennedy, 2008), stimulus-response learning between provocative events and impulsive-RA (Stahl, 2015), or mediating enhanced reward sensitivity in RA populations in general is not clear yet (Buckholtz et al., 2010).

Previous PSAP neuroimaging studies were either not designed to specifically test effects of RA on reward-related vs. retaliation-related brain responses (New et al., 2009; Schluter et al., 2013), or did not find any effects (Kose et al., 2015). Here, using the fMRI-adapted PSAP version by Kose et al. (Cherek, 1981; Kose et al., 2015), we compared the neural reactivity for two competing motivated behaviors, monetary reward-seeking vs. retaliatory behavior, between reactive aggressive men (RA, meeting criteria for full or subclinical IED) vs. non-aggressive controls. Consistent with the idea that the propensity for RA is linked to enhanced reward sensitivity (Buckholtz et al., 2010; Stahl, 2015) including enhanced reward-seeking behavior (Kose et al., 2015), we predicted that RA individuals will show increased reward-seeking behavior and increased activation of the mesocorticolimbic salience network when engaging in monetary-reward-seeking vs. retaliation-driven behavior. We further hypothesized that increased retaliatory behavior in RA individuals vs. controls would be linked to decreased retaliation-related activation in prefrontal “top-down control” areas (Kose et al., 2015) and to increased retaliation-related activation in mesolimbic “bottom-up drive” areas (cf., Siever, 2008) including the striatum (Krämer et al., 2011; Beyer et al., 2014; Gan et al., 2015).

## Methods and materials

### Participants

Healthy individuals and individuals having “anger issues” were recruited from the general population, through newspaper advertisements and word-of-mouth. All individuals provided written informed consent prior to study participation in accordance with Stony Brook University's Institutional Review Board.

All participants underwent psychiatric interviewing including the Structured Clinical Interview (Ventura et al., 1998) to assess DSM-IV Axis-I psychiatric disorders and Axis-II Cluster-B personality disorders including antisocial and borderline personality disorder (First et al., 1997). Significant behavioral features consistent with IED were assessed based on an interview (Coccaro et al., 2004; Coccaro, 2012). In line with the “Research Domain Criteria” approach for functional domains such as negative valence systems (e.g., reactivity to provocation) (Morris and Cuthbert, 2012), we included RA participants with full (IED-interview score > = 15) and subclinical IED (operationalized in the current study as an IED-interview score = 13–14) to acknowledge the continuous nature of aggressive behavior. The State-Trait Anger Expression Inventory-2 (STAXI-2, Spielberger, 1988) was used to validate the grouping of participants on trait anger and anger expression.

Eleven male RA individuals (*n* = 6 full IED, *n* = 5 subclinical IED), and 14 healthy male controls without any DSM-IV psychiatric disorder performed four runs of the fMRI-PSAP. Of those, 18 participants (9 RA, as confirmed by elevated trait anger and anger expression scores on the STAXI relative to 9 non-aggressive controls, Table 1) were included in the final fMRI analysis (see Data analysis for fMRI inclusion criteria). Groups were matched on age, race, handedness, years of education, and estimates of verbal intelligence (Reading scale, Wide Range Achievement Test-III, Wilkinson, 1993), but not on non-verbal intelligence (Matrix reasoning scale, Wechsler Abbreviated Scale of Intelligence, Wechsler, 1987) and current depression symptoms (Beck and Steer, 1984; Table 1). No group differences were observed in current, past or occasional alcohol, cigarette or marijuana use based on the Structured Clinical Interview (Pearson Chi-square tests, *p* > 0.10). Four RA individuals reported comorbid disorders which are common for IED (Coccaro et al., 2005; Kessler et al., 2006): *full IED*, current general anxiety disorder and a single/remitted major depressive episode (*n* = 1), remitted cannabis abuse (*n* = 1); *subclinical IED*, remitted alcohol abuse (*n* = 1), current & lifetime antisocial personality disorder (*n* = 1).

**Table 1:** **Demographics (age, ethnicity, education), handedness, estimates of verbal, and non-verbal intelligence, depressive symptoms, and self-report anger measures for the reactive aggressive (RA) vs. control group**.

**Participants (all male)**	**RA (4 full IED, 5 sub-clinical IED)**	**Controls (*n* = 9)**	**Test statistic**	***p*-value**
Age (years)	34.4 ± 7.5	31.8 ± 6.5	^*t*^(16) = −0.81	*p* = 0.430
Ethnicity (Black/Hispanic/Caucasian)	5/4/0	4/3/2	*χ^2^* = 0.225	*p* = 0.324
Education (years)	12.9 ± 1.4	13.7 ± 1.3	^*t*^(16) = 1.12	*p* = 0.285
Handedness (right/left)	9/0	8/1	*χ^2^ = 1.06*	*p* = 0.303
Non-verbal intelligence: Wechsler Abbreviated Scale of Intelligence—Matrix Reasoning^**^	7 ± 3.2	12.1 ± 2.0	*U* = 6.5, *n* = 17	***p*** = **0.004**
Verbal intelligence: Wide Range Achievement Test III—Reading Scale (grade equivalent)	10.1 ± 3.6	12.5 ± 0.5	*U* = 24, *n* = 17	*p* = 0.277
Depressive Symptoms (BDI)^*^	7.6 ± 5.4	2.7 ± 3.4	*U* = 65, *n* = 18	***p*** = **0.031**
Trait anger (STAXI-2)^***^	23.2 ± 5.4	12 ± 3.8	^*t*^(16) = −5.12	***p*** < **0.001**
Anger expression out (STAXI-2)^**^	21.4 ± 5.1	14.2 ± 4.0	^*t*^(16) = −3.34	***p*** = **0.004**

*Estimates of non-verbal and verbal intelligence were available for 15 participants (missing data for n = 1 RA [non-verbal], and n = 1 control [verbal])*.

* *p < 0.05*,

** *p < 0.01*,

*** *p < 0.001; Independent t-tests were applied to test for group differences of normally and Mann-Whitney U-tests to test for group differences of non-normally distributed variables*.

*The bold values indicate significant differences between groups*.

Exclusion criteria for study participation were any (a) neurological disease including seizures, history of head trauma with loss of consciousness (>30 min); (b) major medical conditions (e.g., cardiovascular, endocrinological, oncological, or autoimmune diseases); (c) major psychiatric disorder with psychosis (e.g., schizophrenia, bipolar disorder); (d) use of psychoactive medication within 6-months prior to study date; (e) positive urine screens for psychoactive drugs or their metabolites (amphetamine/methamphetamine, cocaine, phencyclidine, benzodiazepines, cannabis, opiates, barbiturates, inhalants); and (f) MRI contraindications.

### fMRI-PSAP

Participants were told that our study examined mood, social interaction, and motor coordination (avoiding explicitly the terms “aggression” or “competition”). Participants were instructed that they and another male “person” (located in a different building) could earn as much money as possible by pressing buttons. The other person had the opportunity to subtract $1 from the participant's earnings at any time during the test and add this money to his own earnings; thereby provoking retaliatory behavior. In contrast, participants could subtract $1 from the other person but could not add this money to their earnings (thus choosing to forgo monetary gain).

**Figure 1:**
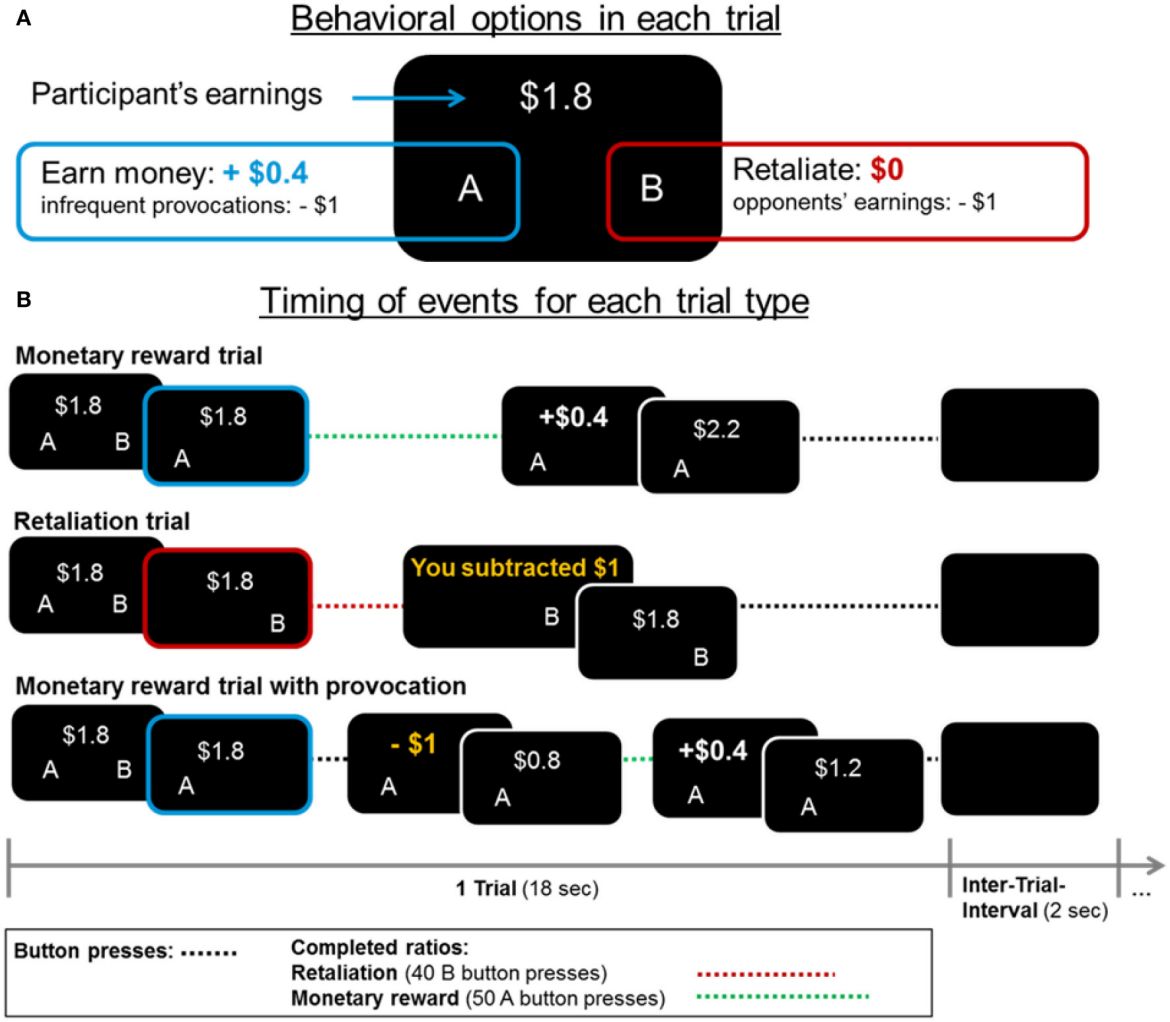
**(A)** In each fMRI-PSAP trial, participants could choose between increasing their earnings by $0.4 (option “A”) and retaliating by subtracting $1 from their opponent's earnings (option “B,” no monetary gain to participant). **(B)** Events and earnings/losses are displayed for each trial type. Each trial lasted for 18 s. Within a trial participants could not switch between the monetary and retaliatory option. However, they could complete multiple monetary (=50 button presses) or retaliatory ratios (=40 button presses) if they were fast enough. There was a 2-s interval between trials (Inter-trial-interval) in which a blank screen was presented.

Each fMRI-PSAP run lasted for 363 s including 18-s trials and 2-s inter-trial intervals. At the beginning of each trial, the accumulated earnings were presented and participants had the choice between increasing their earnings by $0.4 by pressing the A-button 50 consecutive times with their left thumb (1 monetary ratio = 50 A-button presses), or subtracting $1 from their opponent's earnings by pressing the B-button 40 consecutive times with their right thumb (1 retaliatory ratio = 40 B-button presses; see Figure 1). Within each 18-s reward or retaliation trial, participants could press the respective button chosen at the beginning of the trial as often as possible to complete as many monetary or retaliatory ratios; they could not switch buttons within a trial. Participants were unaware that the opponent was fictitious and that the subtractions of money (provocations) occurred at random intervals (6–60 s between provocations) in the monetary trials (compare, Kose et al., 2015). There were no provocations in retaliation trials.

Compared to previous neuroimaging and behavioral PSAP studies (Cherek et al., 1991; New et al., 2009; Perez-Rodriguez et al., 2012; Schluter et al., 2013; Kose et al., 2015), we adjusted the effort of obtaining a monetary reward with achieving retaliation (i.e., 50 A- vs. 40 B-button presses to complete a ratio) to keep both effort and motor-related brain activation comparable across reward and retaliation trials. In agreement with the original behavioral PSAP literature and the Kose et al. (2015) study, which used a comparable fMRI-adapted PSAP, we used the number of monetary and retaliatory responses (A- and B-button presses) as our main outcome variables as they are most meaningful in indicating how hard individuals worked to complete the respective ratios. Additionally, the “number of provocations experienced” and total “earnings” were assessed. Other PSAP aggression measures (e.g., #retaliatory responses/#provocations, or #retaliatory responses/#monetary responses) are not reported here, but yielded similar results.

Before and after the fMRI-PSAP, all participants rated their positive and negative affect using the Positive and Negative Affective Schedule (Watson et al., 1988). Moreover, we asked participants after each run how they felt about their opponent and about their strategy at the end of the PSAP. Based on these self-report measures, all participants believed in the deception, were engaged in the game, were competitive and reported to be annoyed by the subtractions of money.

### Data analysis

#### fMRI-PSAP behavior

We assessed behavioral effects on the number of monetary and retaliatory responses, earnings and number of provocations (a) for the single fMRI-PSAP run for which brain responses were analyzed (for details, see fMRI data analysis) using four independent *t*-tests and (b) across all runs using four separate 4 (fMRI runs) × 2 (Group: RA vs. controls; *n* = 1 RA participant excluded from analysis because of only three runs) repeated-measures ANOVAs.

#### fMRI data acquisition and statistical analysis

Functional MRI was performed on a 4T whole-body Varian/Siemens MRI scanner. The blood oxygenation level dependent (BOLD)-fMRI contrast was measured with a T2^*^-weighted single-shot gradient-echo planar imaging sequence; Echo time = 20 ms, repetition time = 1600 ms; 3.125 × 3.125 mm^2^ in-plane resolution; 4 mm slice thickness; 1 mm gap; 33 coronal slices; Field of view = 200 mm; 64 × 64 matrix size; 90°-flip angle; 200 kHz bandwidth with ramp sampling, 4 dummy scans. Padding, earplugs, and headphones were used to minimize scanner noise (Tomasi et al., 2005). In each fMRI-PSAP run, 227 volumes of 33 coronal slices were acquired.

##### Preprocessing

fMRI data were preprocessed and analyzed with statistical parametric mapping (SPM8, Wellcome Trust Centre for Neuroimaging, London, UK). For preprocessing, fMRI data were realigned, unwarped, normalized to the standard MNI-EPI template, and smoothed with an 8-mm FWHM isotropic Gaussian kernel. The resampled voxel size was 2 × 2 × 2 mm. Prior to first-, and second-level analyses with SPM8, quality criteria for the inclusion of fMRI-PSAP runs into the fMRI analysis were specified following Kose et al. (2015): free of imaging artifacts or extreme head motion (>3.75 mm/degree of translation/rotation); and with at least 2 monetary ratios, 2 retaliatory ratios, and 2 provocations experienced, with these events spaced relatively evenly within the run to avoid bias from low frequency drifts of the BOLD signal over time. Based on these criteria, seven participants (*n* = 2 IED, *n* = 5 controls) were excluded from final fMRI analysis because estimation of retaliation-related brain responses was not possible due to less than two retaliatory ratios in any one of the runs. For the remaining 18 participants, brain responses were analyzed for one out of four fMRI-PSAP runs (run1/run2/run3/run4, *n* = 14/1/3/0, no significant group difference, Pearson Chi-square test, *p* > 0.05) to keep the number of runs comparable across participants as for some participants only one run met all these criteria.

##### First-level analysis

For each participant, individual brain responses were modeled as blocks of monetary responding (1 block = 1 monetary ratio, 50 A-button presses), separating between monetary blocks with and without provocations, and blocks of retaliatory responding (1 block = 1 retaliatory ratio, 40 B-button presses) using a general linear model. Boxcar functions corresponding in length to the duration of monetary and retaliatory blocks (dependent on individual response speed) were convolved with SPM8's canonical hemodynamic response function. On average, participants completed 11.7 monetary ratios (±SD 2.5), 6 retaliatory ratios (±SD 3.1), and experienced 2.6 provocations (±SD 0.7) within the analyzed fMRI run. Although, the number of reward-seeking responses varied between participants across all four runs, it is important to note that, within the single fMRI run we modeled there were no group differences in the number of modeled events (monetary, retaliatory and provocation; independent *t*-tests: *p*_s_ > 0.30). Functional MRI time series were high-pass filtered (1/288 Hz cutoff) to remove low frequency drifts (we did not use the default of 1/128 Hz due to relatively small number of events per condition in some participants). The parameter for each condition was estimated using the general linear model at each voxel. For each participant, we defined activation as the contrast between the parameter estimates for “Reward (no provocation) vs. Retaliation” to focus on neural circuits involved in reward-seeking vs. retaliatory behavior. However, we did not compute contrasts for “Reward” and “Retaliation” separately as there was no adequate baseline in the current fMRI-PSAP design. Due to a low number of provocations in the single fMRI run, we did not explore provocation-related brain activation.

##### Second-level analysis

We compared brain responses between groups by entering one contrast image [“Reward (no provocation) vs. Retaliation”] per subject into the SPM8 Random Effects procedure (including a non-sphericity correction for possible between-group heterogeneity of variance) and applying independent *t*-tests: (a). “RA>Controls” and (b). “RA<Controls.” Additionally, we report differences in activation for “Reward>Retaliation” and “Reward<Retaliation” across groups. Moreover, to investigate if reward-related brain responses (“Reward>Retaliation”) in the single fMRI run were associated with group differences in reward-seeking behavior (i.e., #monetary responses) observed across all fMRI runs, we computed a brain-behavior voxelwise regression analysis across the whole-brain using SPM8. All whole-brain voxelwise analyses were performed using a corrected cluster-level false positive rate of *p* < 0.05 with at least 86 connected voxels (volume = 688 mm^3^) determined by simulating fMRI activation based on the given imaging and preprocessing parameters using 1000 Monte-Carlo simulation iterations (Slotnick et al., 2003) (http://www2.bc.edu/~slotnics/scripts.htm). The corrected cluster-level threshold was applied to an initial uncorrected voxel-wise threshold of *p* < 0.005. Based on Buckholtz et al. (2010), we additionally performed region of interest (ROI) analyses in SPM8 with two 10-mm spheres in the right and left ventral striatum (MNI coordinates: *x* = ±12, *y* = 10, *z* = −6; center coordinates were based on a meta-analysis on reward processing Liu et al., 2011, not on our own results) using a family-wise error corrected significance threshold of *p* < 0.05. For display purposes, we extracted the percent of whole-brain BOLD signal for each participant's “Reward>Retaliation” contrast for each significant cluster of the whole-brain voxelwise analyses using MARSBAR (http://marsbar.sourceforge.net/).

Non-verbal intelligence, indicated based on the Matrix Reasoning scale of the Wechsler Abbreviated Scale of Intelligence (Wechsler, 1987), and depressive symptoms, which both differed between groups, did neither affect behavioral variables nor activation within brain regions showing group differences for “Reward vs. Retaliation” or brain-behavior correlations and were therefore not included as covariates in statistical analyses.

## Results

### fMRI-PSAP behavior

**Figure 2:**
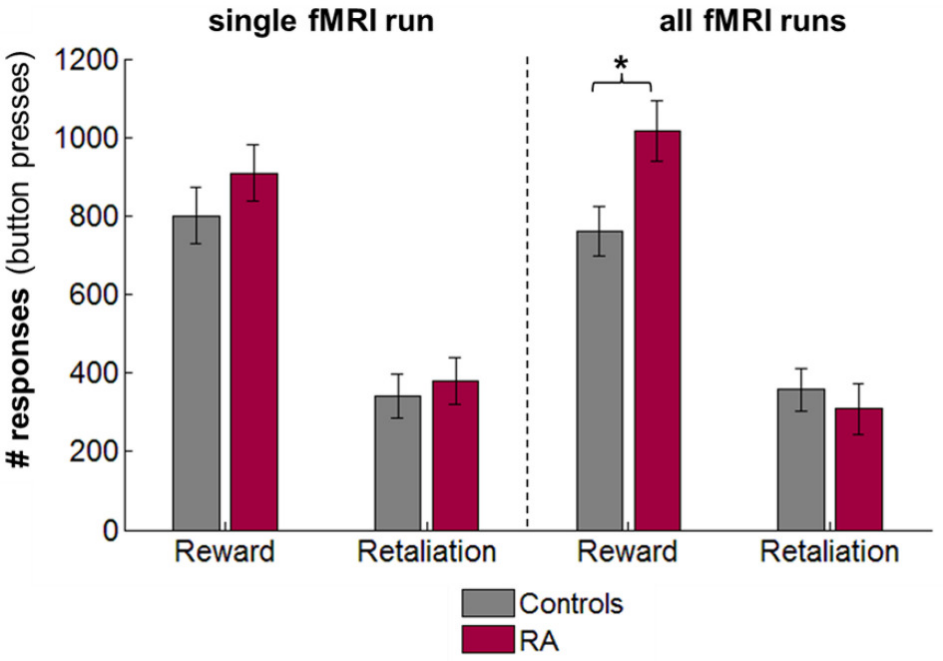
**Retaliatory and monetary reward-seeking responses displayed for reactive aggressive participants (RA) and controls for the single fMRI run for which brain responses have been analyzed (left), and mean responses across the four fMRI PSAP runs (right; responses were collapsed across runs as there was no effect of run and run x group interaction on behavioral responses)**. There were no significant differences between groups for the single fMRI run. Averaged across the four fMRI runs, RA individuals worked significantly more than controls to earn money, but not to retaliate. Abbreviations: fMRI, functional-magnetic resonance imaging; n.s., not significant; PSAP, Point-subtraction aggression paradigm. ^*^p < 0.05.

In the single fMRI run, there were no group differences in number of monetary and retaliatory responses (Figure 2 left), earnings, and provocations (independent *t*-tests, *p*_s_> 0.30). Across all 4 runs, the 4 × 2 repeated-measures ANOVAs revealed that RA participants pressed significantly more buttons for the monetary reward than controls [main effect of group, *F*_(1, 15)_ = 7.62, *p* = 0.015, partial η^2^ = 0.34], but the number of retaliatory button presses did not differ between groups [*F*_(1, 15)_ = 0.15, *p* = 0.701, partial η^2^ = 0.01; Figure 2 right]. There was no significant effect of run on either the number of monetary [*F*_(3, 45)_ = 1.35, *p* = 0.27, partial η^2^ = 0.08] or retaliatory responses [*F*_(3, 45)_ = 1.38, *p* = 0.261, partial η^2^ = 0.08], and no significant run × group interaction [reward: *F*_(3, 45)_ = 2.61, *p* = 0.063, partial η^2^ = 0.15; retaliation: *F*_(3, 45)_ = 2.35, *p* = 0.085, partial η^2^ = 0.14]. While RA individuals did not earn overall more money than controls [*F*_(1, 15)_ = 1.01, *p* = 0.33, partial η^2^ = 0.06; no effect of run], a significant run × group interaction indicated a rise in earnings over the four runs in RA participants, and a decrease in controls [*F*_(3, 45)_ = 3.28, *p* = 0.03, partial η^2^ = 0.18]. However, *post-hoc* independent *t*-tests did not reveal any significant difference between groups for earnings in any one of the runs (*p*_s_ > 0.05). Additionally, RA individuals experienced significantly more provocations across runs [*F*_(1, 15)_ = 7.53, *p* = 0.015, partial η^2^ = 0.33; no effect of run or run × group interaction], possibly because RA individuals chose more often the monetary option (provocations occurred in monetary trials only).

There were no differences in positive and negative affect pre and post the fMRI-PSAP across all participants (Wilcoxon Signed Rank tests; *p*_s_ > 0.046, corrected α-level: *p* < 0.0025, 20 scales), and between groups (for pre and post scores separately, and pre-post difference scores, Mann Whitney-U tests; *p*_s_ > 0.11).

### Reward- vs. retaliation-related brain responses

**Figure 3:**
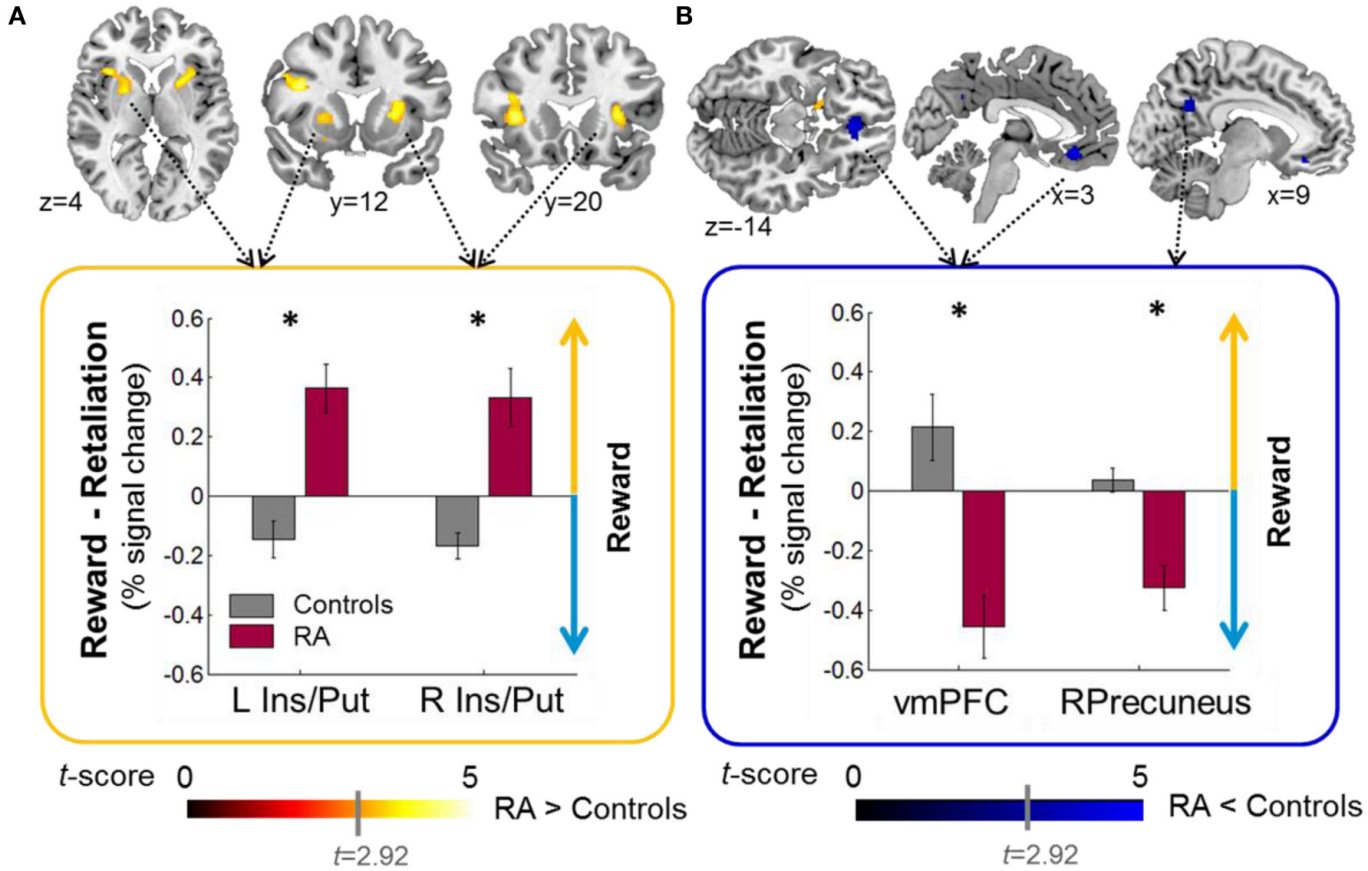
**Group effects on brain activity during sustained button pressing to gain money (reward) compared to achieving retaliation for a single fMRI-PSAP run (see Methods, fMRI preprocessing for reasons for including only one out of four fMRI-PSAP run into analysis). (A)** RA individuals relative to controls exhibited increased activation within brain areas of the mesocorticolimbic salience network including the bilateral anterior insula and putamen, when working to obtain a reward vs. achieving retaliation (RA>controls; warm color scale). **(B)** RA individuals relative controls exhibited decreased activation within brain areas of the default-mode network, the bilateral vmPFC, and the Precuneus, when working to obtain a reward vs. achieving retaliation (RA<Controls, blue color scale). In the bar plots, the yellow upward pointing arrow indicates that brain responses were increased for reward vs. retaliation, and the blue downward pointing arrow indicates that brain responses were decreased for reward vs. retaliation. Whole-brain results are significant at a corrected cluster-threshold of *p* < 0.05 with at least 86 connected voxels (initial uncorrected height threshold: *p* < 0.005). Abbreviations: < 0.001, ^*^p < 0.05, Ins, insula; L, Left; n.s., not significant; Precun, precuneus; Put, putamen; *r*, Spearman's correlation coefficient; R, Right; RA, reactive aggressive group; vmPFC, ventromedial PFC.

Relative to controls, when working for reward vs. retaliation, RA individuals exhibited increased brain acitivity in the mesocorticolimbic salience network (bilateral: anterior insula, putamen; Left: inferior frontal operculum; RA>Controls, Figure 3A, Table 2) and decreased activation in areas belonging to the default-mode network (bilateral ventro-medial [vm] PFC, right precuneus; RA<Controls, Figure 3B, Table 2). Across groups, brain responses were increased for reward vs. retaliation in the left cuneus (Reward>Retaliation) and decreased in the precuneus, the superior frontal gyrus, and the occipital cortex (Reward<Retaliation, Table 2). None of the ROI analyses revealed significant differences between groups nor between reward and retaliation.

**Table 2:** **Whole-brain results of group comparisons for Reward vs. Retaliation**.

**Brain Area**		**BA**	**MNI coordinates**			**Cluster size**	**Peak *T***	***P*** **uncorrected**	
			***x***	***y***	***z***	***k***		**Cluster-level**	**Peak-level**
**REWARD**>**RETALIATION ACROSS GROUPS**									
L	Cuneus	18	−20	−90	14	92	4.51	0.196	<0.001
**REWARD**<**RETALIATION ACROSS GROUPS**									
R	Calcarine	18	2	−98	6	174	5.25	0.083	<0.001
L	Superior Frontal Gyrus	9	−18	42	42	318	4.59	0.024	<0.001
L	Superior Frontal Gyrus	9	−28	52	36		4.54		<0.001
L	Superior Frontal Gyrus	8	−14	42	50		4.00		<0.001
L	Lingual Gyrus	19	−18	−54	−6	421	4.34	0.011	<0.001
L	Lingual Gyrus	18	−16	−76	−8		4.00		<0.001
L	Cerebellum, Declive		−14	−78	−16		3.55		0.001
L	Precuneus	7	−10	−62	46	378	4.20	0.015	<0.001
L	Precuneus	31	−8	−48	44		3.47		0.002
R	Precuneus	7	8	−56	32		3.40		0.002
**RA**>**CONTROLS, REWARD**>**RETALIATION**									
R	Insula/Putamen	−	30	16	10	214	4.76	0.057	<0.001
L	Insula	13	−36	20	6	608	4.25	0.003	<0.001
L	Inferior frontal operculum	46	−40	12	24		4.22		<0.001
L	Putamen	−	−22	6	0		4.03		<0.001
**RA**<**CONTROLS, REWARD**>**RETALIATION**									
bl	vmPFC	11	0	36	−12	142	4.78	0.113	<0.001
R	vmPFC/Gyrus rectus	25	10	34	−14		3.71		0.001
R	Precuneus	31	14	−64	28	123	4.24	0.138	<0.001
R	Precuneus/PCC	31	8	−58	30		3.67		0.001

*Abbreviations: bl, bilateral; L, Left; PCC, Posterior cingulate cortex; R, right; RA, reactive aggressive; vmPFC, ventro-medial prefrontal cortex*.

### Brain-behavior regression

**Figure 4:**
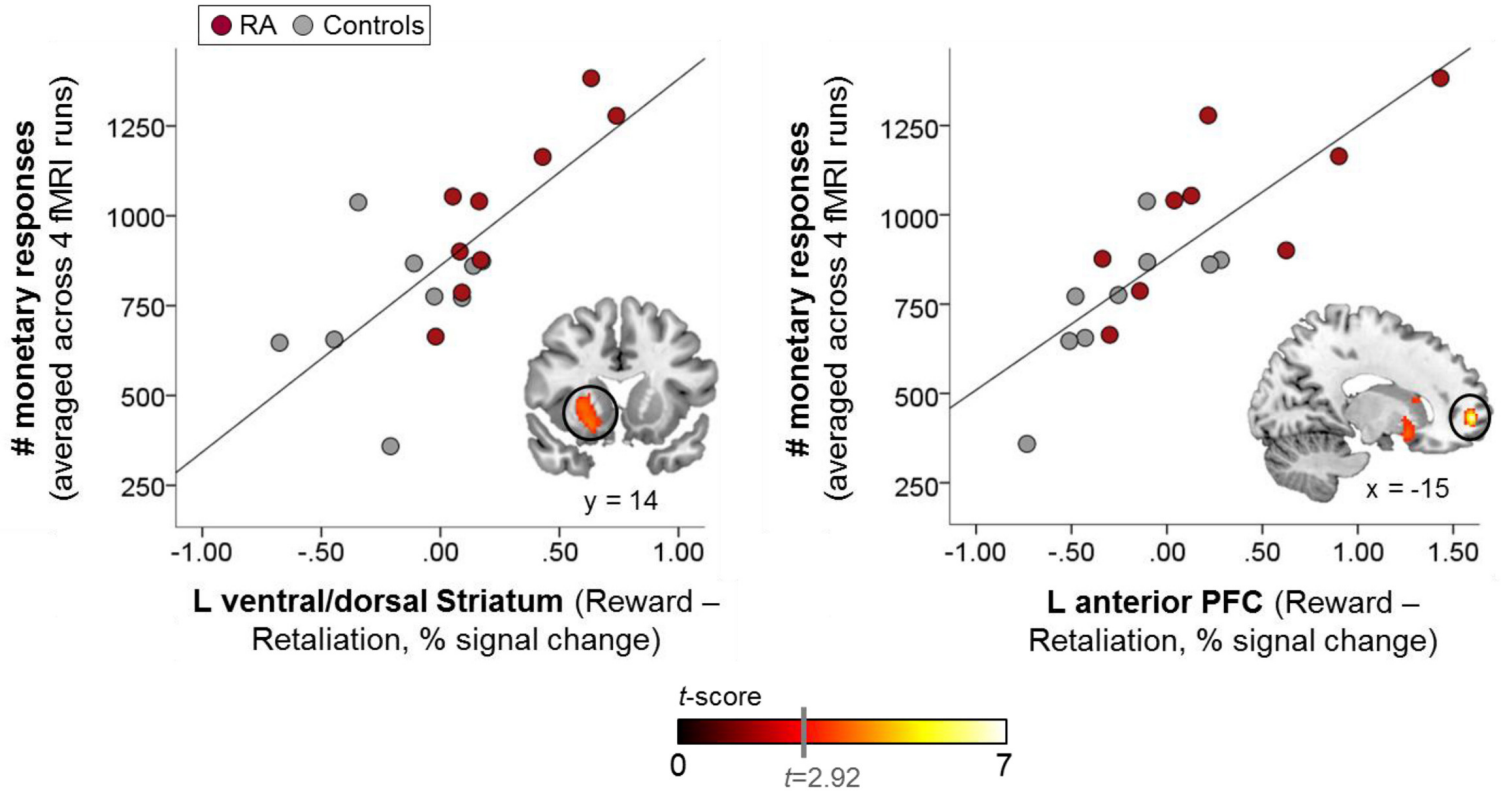
**Whole-brain-behavior correlations: brain reactivity in the left ventral/dorsal striatum, and the left anterior prefrontal cortex (PFC) to reward vs. retaliation in the single fMRI run was positively correlated with reward-seeking behavior (i.e., monetary responses) across all fMRI runs, which was significantly increased in the RA group (warm color scale)**. The more pronounced the brain response to reward relative to retaliation within these mesocorticolimbic areas, the more increased the overall reward-seeking behavior. Whole-brain results are significant at a corrected cluster-threshold of *p* < 0.05 with at least 86 connected voxels (initial uncorrected height threshold: *p* < 0.005).

The whole-brain voxelwise regression analysis (Table 3) revealed significant positive correlations in the left ventral/dorsal striatum and the anterior PFC (Figure 4), and significant negative correlations in the precuneus, the supramarginal gyrus, and medial and lateral PFC for reward-related brain responses (“Reward>Retaliation”) of the single fMRI run and monetary responses across all four runs. ROI analyses supported the whole-brain results in the striatum, revealing a significant positive correlation in the left ventral striatum (MNI coordinates: *x* = 20, *y* = 14, *z* = −10; cluster size = 7 voxels; *T* = 3.81; family-wise error corrected: *p*_peak_ = 0.041, *p*_cluster_ = 0.031). There was no difference in correlations between groups.

**Table 3:** **Whole-brain correlations between brain responses for Reward vs. Retaliation of the single fMRI run and overall reward-seeking behavior across all four fMRI runs**.

**Brain Area**		**BA**	**MNI coordinates**			**Cluster size**	**Peak *T***	***P*** **uncorrected**	
			***x***	***y***	***z***	***k***		**Cluster-level**	**Peak-level**
**POSITIVE CORRELATION**									
L	vmPFC	10	−16	58	0	203	6.85	0.060	<0.0001
L	Putamen, Ltf. Nucleus	−	−20	14	−10	466	3.81	0.008	<0.001
L	Insula	13	−30	20	6		3.78		<0.001
L	Putamen, Ltf. Nucleus	−	−24	14	0		3.72		<0.001
**NEGATIVE CORRELATION**									
L	Postcentral Cyrus	40	−62	−26	22	288	6.42	0.029	<0.0001
L	Precentral Gyrus	4	−62	−14	40		3.34		0.002
R	dorso-medial PFC	8	8	48	46	153	5.46	0.098	<0.0001
L	dorso-medial PFC	9	−4	52	42		3.47		0.002
R	Precuneus	31	10	−66	28	455	5.22	0.008	<0.0001
R	Precuneus	7	4	−58	32		4.14		<0.001
L	Precuneus	7	−6	−68	40		3.60		0.001
L	Superior Frontal Gyrus	8	−16	48	52	93	4.70	0.188	<0.001

*Abbreviations: Ltf., lentiform; PFC, prefrontal cortex*.

## Discussion

When given a choice between two competing options, accumulating money vs. retaliating for provocations (i.e., subtractions of money), men with clinically pronounced RA compared with controls exhibited an up-regulation of the insular-striatal area of the mesocorticolimbic salience network, and a down-regulation of the default-mode network while working for a monetary reward vs. retaliation in a single fMRI-PSAP run. Further, while RA individuals and controls did not differ in their retaliatory and reward-seeking behavior in this single fMRI-PSAP run, RA individuals showed overall increased reward-seeking behavior across all four fMRI-PSAP runs (i.e., number of monetary responses). Whole-brain correlation analyses across all participants revealed that increased reward-related reactivity of the left ventral/dorsal striatum and the anterior PFC in the single fMRI run was associated with the overall increased motivation to work for money across all four fMRI-PSAP runs.

### Effects of RA in the mesocorticolimbic salience network

Increased activation of the anterior insula and the putamen during a single run, along with pronounced reward-seeking behavior in RA individuals across all runs likely points to enhanced salience attribution to effortful-behavior with the goal of obtaining money (Liu et al., 2011) vs. achieving retaliation. The anterior insula and putamen belong to the mesocorticolimbic salience network and are anatomically and functionally closely connected (Postuma and Dagher, 2006). Both areas respond to negative and positive rewards suggesting their role in salience- rather than value-processing (Liu et al., 2011; Bartra et al., 2013). Being part of an intrinsic connectivity network that links cognition, emotion, and interoception (Laird et al., 2011), the anterior insula plays a prominent role in salience processing in attention-demanding situations (Seeley et al., 2007; Craig, 2009, 2011; Touroutoglou et al., 2012). For the putamen, we observed the increased reward-related activation for “RA>controls” in the pre-commissural part of the dorsal striatum (Draganski et al., 2008), a region implicated in processing the salience of stimuli (Bromberg-Martin et al., 2010; Bartra et al., 2013), in stimulus-action-outcome learning, and action selection based on reward expectations (Haruno and Kawato, 2006; Balleine et al., 2007; Bromberg-Martin et al., 2010). In the current fMRI-PSAP, the contingencies of stimulus-action-outcome learning were simple; participants could either opt for the reward or the retaliation, with approximately equal effort for each option. Interestingly, whole-brain and ROI regression analyses showed that pronounced reward-related activity in the ventral/dorsal striatum (and the anterior PFC), observed in the single run, was associated with overall increased reward-seeking behavior across all runs and all participants. Our findings are consistent with previous reports that increased reactivity of the mesocorticolimbic system during the anticipation of rewards predicts the average effort invested to obtain monetary rewards (Bühler et al., 2010), and support the notion that increased reactivity of the brain's reward system might be a neural marker for impulsive RA (Buckholtz et al., 2010; Kose et al., 2015; Stahl, 2015). Thus, increased reward-related striatal activity in RA individuals likely reflects enhanced salience attribution to monetary rewards, and possibly early habit formation to seek rewards instead of retaliation in our specific experimental situation. In line with Buckholtz et al. (2010), our data indirectly support Stahl's theory that impulsive retaliatory aggression might be a “compulsive habit” by showing that RA individuals share enhanced reward sensitivity with other “compulsive” problem behaviors such as drug and behavioral addiction (Stahl, 2015). However, due to task design limitations (see below), we could not test group differences separately for reward-seeking and retaliatory behavior, which would have been interesting as both conditions may share appetitive features indicated by striatal responsivity observed for the anticipation of monetary rewards (Liu et al., 2011) and the engagement in retaliation/aggression (Krämer et al., 2011; Beyer et al., 2014; Gan et al., 2015). Thus, it remains to be elucidated if recurrent impulsive-RA develops due to a shift from ventral striatum activity triggering goal-directed behavior to more habit-like stimulus-directed behavior controlled by the dorsal striatum (Stahl, 2015), as previously proposed for compulsive drug use behavior (Everitt and Robbins, 2005).

### Effect of RA in the default-mode network

Interestingly, we observed decreased precuneus and vmPFC activation for reward vs. retaliation in the RA group compared to controls. Down-regulation of the precuneus and vmPFC in RA individuals during reward-seeking vs. retaliation-driven behavior could indicate increased suppression of the default-mode network which is deactivated during attention-demanding cognitive tasks and active at rest (Raichle et al., 2001; Fox et al., 2005; Utevsky et al., 2014). Previously, we proposed that increased metabolic activity of the default-mode network at rest in aggressive vs. non-aggressive individuals might be a neural marker of increased self-referential processing (Alia-Klein et al., 2014). Here, we suggest that the down-regulation of the default-mode network during reward-seeking behavior in RA individuals might indicate that they were more engaged in the reward condition than controls. As previously shown for neuropsychiatric disorders (e.g., schizophrenia and biploar disorder, Whitfield-Gabrieli and Ford, 2012), there might be compromised functioning of the default-mode network in human RA.

It is important to note that the group differences observed in the mesocorticolimbic areas including the putamen and the anterior insula and in areas of the default-mode network including the precuneus and the vmPFC in the single fMRI run occurred in the absence of group differences in behavior and number of provocations in this single run. Thus, group differences in brain activation cannot be explained by an imbalance in the number of modeled events in the first-level GLMs between groups.

### Implications

Pronounced reward-seeking behavior and up-regulation of the mesocorticolimbic salience network during reward-seeking behavior in RA individuals imply that the use of positive reinforcement (e.g., contingency management) could be beneficial in anger management treatments. According to a previously proposed theory on “the behavioral economics of violence” (Rachlin, 2004), violent behavior is regulated because it is costly in the long run (e.g., dropping out of school, lower education and lower income jobs; health-related problems). However, Rachlin argues that the short-term benefits (e.g., releasing anger) can outweigh the short-term costs of violent behavior (e.g., physical effort, injuries), a mechanism potentially involved in severe RA. In real life, the long-term benefits of not being aggressive (i.e., more money, stable relationships) are typically delayed and have a low salience. Thus, to reinforce the salience of those long-term benefits, anger management treatments could offer education/social support or even the opportunity to earn money as incentives for aggressive individuals to refrain from aggressive behavior.

### Limitations

Our findings in a small sample of RA men, of whom half reported significant behavioral features but did not meet the criteria for full IED, require caution in generalizing results to a broader population of IED patients. An important limitation of this study is that only men were studied, and thus we do not know whether similar effects would be seen in women. Future neuroimaging studies including both men and women are needed to further investigate how potential sex differences in hormones and brain structure affect the neural circuitry of reactive aggressive behavior (e.g., de Almeida et al., 2015). Nevertheless, there is reason to speculate that women scoring high on RA would show comparable patterns of enhanced reward-seeking behavior and reward-related brain responses in the fMRI-PSAP as shown for men in the current study. For instance, although men have slightly higher odds ratios to be diagnosed with IED once in their lifetime than women (Kessler et al., 2006), the lifetime prevalence for IED in women is 5.6% (compared to 9.3% in men), and experimental evidence from the behavioral PSAP suggests that women are as likely to show retaliatory behavior as men when they are provoked (Bjork et al., 1997; Dougherty et al., 1999). Moreover, the hypersensitivity of the mesocorticolimbic reward system has been observed for men as well as women with pronounced impulsive anti-social traits (Buckholtz et al., 2010).

In contrast to previous PSAP studies in borderline and alcohol-dependent patients with a propensity for disproportionate RA (New et al., 2009; Kose et al., 2015), the RA group did not differ from controls in retaliatory behavior in the current fMRI-PSAP study. Although, this might seem counterintuitive at first blush, healthy individuals were less likely to retaliate when the effort to achieve retaliation was adapted to the effort to obtain money in the PSAP (Cherek et al., 1992). A systematic manipulation of effort ratios and larger sample sizes could help to elucidate if there is an “effort threshold” needed to motivate RA individuals to choose a non-aggressive reinforcement over a retaliatory option.

In the current fMRI-PSAP, one main limitation is that we could only analyze brain responses for a single run because the number of reward and retaliation events was determined by the participants' choices and turned out to be too small in some runs to effectively model brain responses. Future fMRI-PSAP studies should consider using pre-determined rates of reward and retaliation blocks as well as provocations to maximize the number of events per experimental conditions. However, such experimental modifications might ultimately change the behavior one aims to measure with the PSAP as one unique feature of the PSAP is that individuals are free to decide in each trial if they want to earn money or want to retaliate. Here, to allow the freedom to not retaliate it might be recommended to modify the retaliation condition by giving participants the option to adjust the amount they would subtract from their opponent (from $0 to a maximum amount of about $2). Alternatively, researchers might want to increase the intensity of provocations (e.g., subtracting higher amounts of money, and display “malicious joy” by their opponent) to reach the threshold for self-report of anger and to provoke more retaliatory behavior. Moreover, including an adequate baseline (e.g., sustained button pressing for neither a reward nor retaliation) into the fMRI-PSAP would enable the separate comparison of reward and retaliation blocks. When optimizing the fMRI-PSAP in future investigations, it might also be interesting to incorporate the measurement of behavioral contrast effects (Crespi, 1942) to assess how the motivation to seek rewards is altered in RA individuals when the magnitude of the incentive is varied throughout the experiment.

## Conclusions

In the present study, we demonstrate for the first time that an up-regulation of the mesocorticolimbic salience system along with increased monetary reward-seeking behavior might play a role in clinical human RA, exemplified by the psychiatric condition of IED and high trait anger. Up-regulation of the mesocorticolimbic salience system in RA individuals might indicate enhanced salience attribution to monetary rewards vs. retaliation in a situation in which both options require relatively comparable effort. Our findings in the mesocorticolimbic salience system lend empirical support to the idea that maladaptation in the brain's reward system might be a neural substrate of impulsive-RA (Buckholtz et al., 2010; Stahl, 2015). Moreover, down-regulation of the default-mode network in RA individuals during reward-seeking behavior hints at altered functioning of the default-mode network in human RA. Interestingly, this pattern of increased striatal responses, and decreased vmPFC responses, in response to monetary reward is consistent with previous reports in cocaine addiction, another externalizing disorder characterized by poor self-control (Goldstein et al., 2007; Konova et al., 2012). Future studies are needed to examine if impulsive-RA shares neural substrates with addictive behaviors (compare, Moeller et al., 2014; Stahl, 2015). Despite experimental limitations, the current findings emphasize that monetary reward processing and related brain responses are altered in reactive aggressive individuals, entirely consistent with our hypotheses that were derived from prior work (e.g., Buckholtz et al., 2010). Further research with larger sample sizes and optimized experimental designs will help to determine the role of the reward system as a potential target for new treatment options such as contingency management to reduce the occurrence of repeated outbursts of reactive aggression.

## Author contributions

NAK, RZG, SDL, and JLS designed the study. NAK, SJM, MAP, and TM acquired the data. GG, RPC, NAK, SJM, MAP, TM, and JLS contributed to the data analysis. GG, RPC, NAK, SJM, MAP, and RZG interpreted the data. GG wrote the manuscript and included feedback from co-authors into the manuscript. All co-authors reviewed the manuscript for important intellectual content and approved the submission of the final manuscript for publication.

### Conflict of interest statement

The authors declare that the research was conducted in the absence of any commercial or financial relationships that could be construed as a potential conflict of interest.
